# Effects of host extinction and vector preferences on vector-borne disease risk in phylogenetically structured host-hector communities

**DOI:** 10.1371/journal.pone.0256456

**Published:** 2021-08-23

**Authors:** Charles L. Nunn, Alexander Q. Vining, Debapriyo Chakraborty, Michael H. Reiskind, Hillary S. Young

**Affiliations:** 1 Department of Evolutionary Anthropology, Duke University, Durham, North Carolina, United States of America; 2 Duke Global Health Institute, Durham, North Carolina, United States of America; 3 Graduate Program in Animal Behavior, UC Davis, Davis, California, United States of America; 4 INRAE ENVT IHAP, National Veterinary School of Toulouse, Toulouse, France; 5 Department of Entomology and Plant Pathology, North Carolina State University, Raleigh, North Carolina, United States of America; 6 Department of Ecology, Evolution and Marine Biology, University of California, Santa Barbara, California, United States of America; University of Oklahoma Norman Campus: The University of Oklahoma, UNITED STATES

## Abstract

Anthropogenic disturbance impacts the phylogenetic composition and diversity of ecological communities. While changes in diversity are known to dramatically change species interactions and alter disease dynamics, the effects of phylogenetic changes in host and vector communities on disease have been relatively poorly studied. Using a theoretical model, we investigated how phylogeny and extinction influence network structural characteristics relevant to disease transmission in disturbed environments. We modelled a multi-host, multi-vector community as a bipartite ecological network, where nodes represent host and vector species and edges represent connections among them through vector feeding, and we simulated vector preferences and threat status on host and parasite phylogenies. We then simulated loss of hosts, including phylogenetically clustered losses, to investigate how extinction influences network structure. We compared effects of phylogeny and extinction to those of host specificity, which we predicted to strongly increase network modularity and reduce disease prevalence. The simulations revealed that extinction often increased modularity, with higher modularity as species loss increased, although not as much as increasing host specificity did. These results suggest that extinction itself, all else being equal, may reduce disease prevalence in disturbed communities. However, in real communities, systematic patterns in species loss (e.g. favoring high competence species) or changes in abundance may counteract these effects. Unexpectedly, we found that effects of phylogenetic signal in host and vector traits were relatively weak, and only important when phylogenetic signal of host and vector traits were similar, or when these traits both varied.

## Introduction

The hypothesis that anthropogenic disturbance systematically increases disease prevalence has attracted much interest [1, 2]. Although disturbance affects ecological communities in many ways, most empirical and theoretical studies exploring the relationship between disturbance and disease have focused on the likely effects of changes in host species richness resulting from disturbance [3–5]. However, several recent meta-analyses have suggested that, on average, local host species richness may not change strongly even in highly disturbed environments, despite very high rates of species turnover following disturbance [6–10]. For instance, in disturbed environments non-native species or cosmopolitan generalists may persist or increase while native species may decline [e.g., 11, 12], with important impacts on disease dynamics and without any changes in species richness [13]. Indeed, richness of known zoonotic hosts actually appears to increase in disturbed environments [14]. Consistent with these results, studies have shown that realistic changes in species richness following disturbance have very different effects on disease than does natural or randomized effects on richness [15, 16]. Species richness thus has limited utility in understanding these relationships, as it does not capture much relevant information about potential for disease transmission among species in communities.

Metrics of biodiversity that incorporate phylogenetic history are often more strongly impacted by disturbance than is species richness [e.g., 17–19]. This arises partly because closely related species often share traits that make them prone to similar threats; examples of such traits for particular taxa include a slow life history, large or small body size, small geographic ranges, and narrow ecological niches [20–24]. Consequently, risk of extinction or declines in abundance is usually not randomly distributed across species, but instead is often clumped phylogenetically [25, 26]. This phylogenetic clumping has effects on the phylogeny of extant species, as the paired extinction of two sister species prunes the branch to their common ancestor [20]. Thus, on average, extinction of sister species causes greater loss of evolutionary history than loss of less closely connected species, resulting in changes to phylogenetic and functional diversity [17, 27].

Quantifying the impacts of disturbance on phylogenetic diversity is particularly relevant for disturbance-disease relationships because changes in community phylogenetic structure have been linked to disease transmission. For instance, there is now strong evidence for phylogenetic conservatism across host parasite systems, such that the likelihood of a pathogen being shared across host species decreases with increasing phylogenetic distance between hosts [28–30], probably as a result of phylogenetically conserved traits that affect host-pathogen dynamics. Disturbance in particular is thought to drive non-random shifts in ecological communities [15, 31]. As a result, species that have higher average phylogenetic proximity to other species in the community may have higher risk of contracting parasites from heterospecifics (and conversely, phylogenetically distinct species may have lower risks of parasite sharing) [32–34]. Consistent with this, recent work on livestock diseases has shown that incorporating host phylogeny provides better predictive capacity for the incidence of disease than does host richness alone [35]. Likewise, phylogenetic diversity has been shown to predict spatial dynamics of H5N1 outbreaks among wild birds [36]. Increasing recognition of the potential importance of phylogenetic diversity in understanding disease emergence has led to calls for greater research on this topic, including more exploration of the mechanisms by which this relationship may operate [37].

Vector-borne diseases represent a major burden of infectious diseases worldwide, particularly in lower- to middle-income countries in the tropics and sub-tropics [38]. Vector-borne emerging infectious diseases (EIDs) have also been on the rise globally [39]. While many factors, including climate change and environmental perturbation, have contributed to increases in emerging infectious disease [40], changes in host diversity and host composition have repeatedly been hypothesized to drive these patterns [3, 41, 42]. Indeed much of the evidence and theory about diversity-disease relationships has come from vector-borne disease systems, with a focus on host diversity [1, 43].

Five aspects of host-vector relationships are critical to understanding the effects of species loss on vector-borne disease dynamics: host preference, host specificity, host competence, and plasticity in feeding patterns. **Host preference** refers to the preference for particular hosts by vector species, and is a key determinant of vector-borne disease dynamics, with small changes in host preferences shown to considerably affect transmission [44–46]. Notably, specific preferences for different host species in a community vary across vector species [47–49]. This cross-species heterogeneity in host preferences may impact both the timing and intensity of outbreaks [49, 50]. **Host specificity**–the degree to which a vector prefers one versus multiple hosts–is a related concept that is also important for disease transmission in multi-host communities. Vector species that show strong host specificity will drive higher transmission of pathogens within the preferred host, but are less likely to generate spill-over of pathogens between hosts, as compared to vectors with low host specificity (i.e., generalists). Vector-borne disease dynamics are also influenced by variation in competence among host species, where **host competence** refers to the ability of a host to transmit infectious stages of parasites to successfully infect another host or vector [51]. Competence may vary systematically based on the phylogeny of the host and variation in life history or other traits [52]. Another important factor in vector feeding ecology is the degree to which vectors show **plasticity in feeding patterns** when preferred hosts are unavailable [49]. This plasticity represents the degree to which the vector will feed on less preferred hosts when their most preferred hosts is unavailable.

Host preferences of vectors are expected to be linked to host phylogeny because closely related vectors may share traits that facilitate exploitation of host taxa with particular traits [53]. Similarly, closely related host species may share traits (physiological or behavioral) that influence their accessibility or attractiveness to particular vector taxa. Recent studies have found, for example, that more closely related flea species are more likely to be found on more closely related host species [54], and that host communities are compartmentalized, such that certain vectors feed on distinct subsets of closely related host species [55, 56]. These effects could result in phylogenetic clusters of interacting hosts and vectors, which determine disease transmission pathways. Hence, changes in the phylogenetic structure of host and vector communities may also influence disease dynamics in host-vector systems.

Here, we use a theoretical model to investigate whether vector-borne disease transmission is likely to be influenced by phylogenetically-conserved traits of vectors and hosts. One trait in the model also influenced differential extinction of hosts, as might be expected in the context of human activities that differentially impact closely-related organisms; this might happen, for example, if human activities cause higher extinction in larger-bodied organisms or those living in a particular habitat. We expected that the degree of phylogenetic signal in host and vector traits would impact the structure of contacts within host-vector communities, and would thus influence disease transmission. To investigate these expectations, we simulated host-vector communities as bipartite ecological networks, where network nodes represent host species and vector species and edges represent connections generated through simulated vector feeding preferences for simulated host characteristics.

As output from the model, we focused on network structure, aiming to quantify the degree to which interactions are subdivided within the bipartite networks. Networks are often composed of distinct communities, or “modules,” with many edges within subsets of species but fewer edges between those subsets [57]. The degree to which networks are structured can be quantified by network modularity [58], with greater modularity expected to shift the system to increase transmission within modules but reduce disease transmission throughout the system [59, 60]. We focus on modularity over other indices, such as nestedness or centralization, because the epidemiological consequences of modularity are better studied and intuitive: modularity reflects subdivision of a network into more distinct units, which are expected to slow disease transmission in the same way that quarantines reduce spread in human populations. To ensure that changes in modularity reflect changes in transmission [61, 62], we also simulate disease spread on the networks, focusing on speed of transmission.

## Materials and methods

### Modelling strategy

We investigated how host specificity shapes network structure by varying a parameter that influences how strongly a given vector prefers hosts with a given characteristic. Based on how the model was constructed, an increase in host specificity should increase modularity, with vectors focusing on more distinct subsets of hosts, thus strengthening divisions in the host-vector network. By measuring this effect, we were able to validate the model and our analyses, and more importantly, to assess how changes in other variables compare to the effect of changing host specificity (which, based on the model and calculation of modularity, is expected to be among the strongest and most consistent of the effects).

We then investigated how phylogenetic signal in host characteristics and in host preferences among vector species influence network structure. Although anticipated effects here are less obvious than those involving host specificity, we generally expected that stronger phylogenetic signal in host and vector characteristics would result in greater modularity in bipartite host-vector networks, due to stronger connections between closely related hosts that share similar traits and closely related vectors that share similar preferences.

Finally, we investigated how two different aspects of host extinction affect network structure. One set of analyses examined how increasing the rate of random extinction influences network structure, specifically through the effect of a higher extinction rate. Under a birth-death model of diversification of lineages, a higher rate of random extinction (lineage death) will result in a tree with more structure, i.e. more clustering of lineages at the tips of the tree, and long branches connecting those clusters [63–65]. By varying the lineage death rate when generating trees used in our simulations, we thus assessed whether phylogenetic structure influences the structure of host-vector ecological networks. In a second set of analyses, we moved from random extinction of lineages to extinction based on evolved characteristics that also have phylogenetic signal, such as body mass or life history traits. In this case, we removed a proportion of species from a community, with removal based on a quantitative threat trait that we simulated under a Brownian motion evolutionary model on the host phylogeny. We predicted that when the threat trait shows strong phylogenetic signal (resulting in phylogenetically clustered extinctions), measures of modularity would increase after extinction due to loss of phylogenetically clustered host subgroups. (Nee 2001; Nee 2006; Nunn 2011). By varying the lineage death rate when generating trees used in our simulations, we thus assessed whether phylogenetic structure influences the structure of host-vector ecological networks.

To test whether our findings involving modularity have actual disease consequences, we simulated disease transmission on networks that vary in the degree of modularity, predicting a negative association between modularity and speed of disease transmission on the network.

#### The host-vector community phylogenetic model

**Fig 1:**
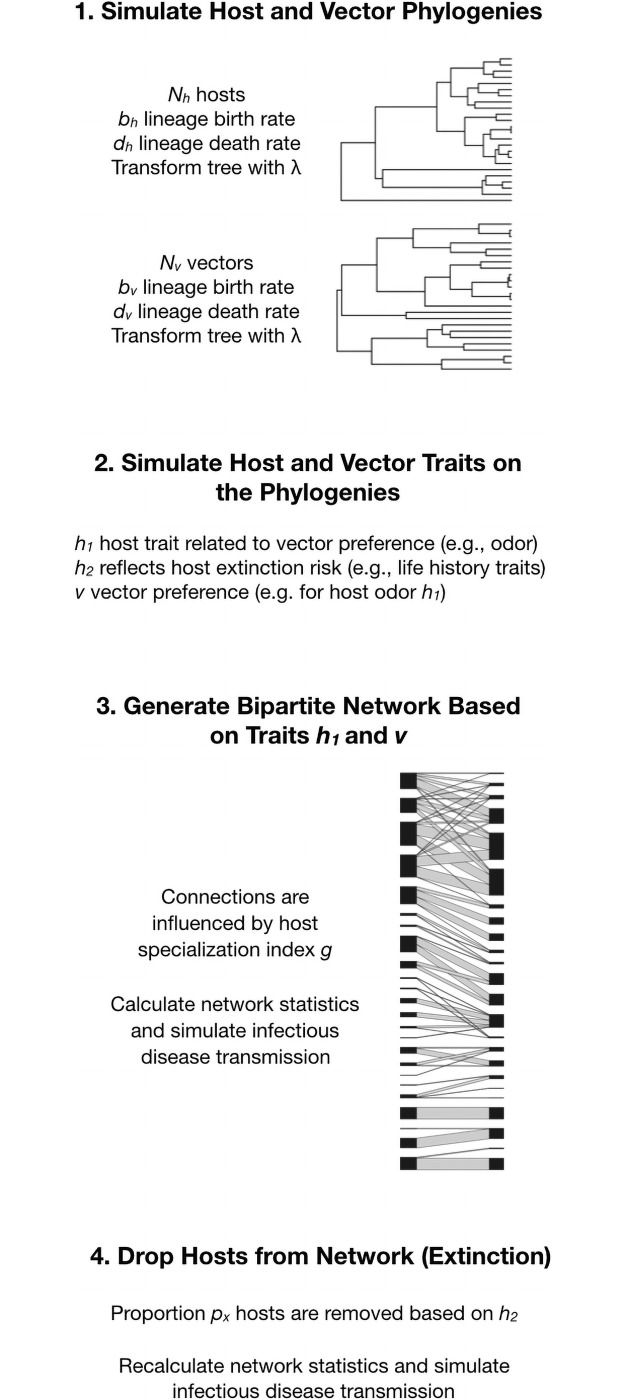
Overview of the modelling approach. The schematic shows the four main steps in the model, labelled 1–4. Note that λ is adjusted to reflect variable phylogenetic signal in the simulated traits, which assumes a Brownian motion model of evolution.

Fig 1 summarizes the main steps in our modeling approach. The host-vector community was represented as a bipartite network. In brief, we simulated traits on phylogenies for hosts and vectors, standardized those traits, obtained distances between the traits, and then used those distances as host-vector edges in a bipartite network. These are weighted edges, not 0’s and 1’s (presence and absence) and based on a mechanistic model of vector preferences for different hosts, under the assumption that these preferences show phylogenetic signal based on shared derived characters in both the hosts and the vectors.

We first generated host and vector phylogenies on which to simulate trait evolution. We assumed that lineages diverge following a birth-death model [66], with new lineages “born” at speciation rate *b* and “dying” at extinction rate *d* [63]. When generating phylogenies, the death rate was not influenced by vector preferences or host phenotypic traits, and all lineages experienced the same probability of speciation and extinction (i.e., rates were constant). To investigate the role of phylogenetic signal in evolved host traits, we transformed trees with the branch length scaling parameter λ [67], which provides a measure of phylogenetic signal reflecting shared evolutionary history of species. In this context, λ is used as a multiplier of internal branch lengths, maintaining a constant root-to-tip distance. Therefore, when λ is zero, all internal branches are multiplied by zero and the tree results in a star phylogeny, representing an absence of phylogenetic signal [68]. Alternatively, when λ is 1, the tree is untransformed, and phylogenetic signal in host and vector traits should be high. Values of λ between 0 and 1 represent intermediate degrees of phylogenetic signal, with higher values resulting in simulated traits that show greater phylogenetic signal.

For every host tree generated, we simulated two independently evolving continuous traits (*h*_*1*_ and *h*_*2*_), based on a Brownian motion model of evolution [65, 68] and values of λ set by the user (ranging from 0 to 1, corresponding to increasing phylogenetic signal), with each trait potentially having a different value of λ and traits simulated independently of one another. We assumed the continuous trait *h*_*1*_ represents a biological host character relevant to vector preferences (e.g., odor characteristics). The second independently evolving host trait (*h*_*2*_) represented a continuous trait, such as body mass or slow life history that make a species more vulnerable to extinction [69, 70]. We likewise simulated another independently evolving trait (*v*) on the vector tree, representing a vector preference for the evolved host characteristics (see below). We assumed that no coevolutionary dynamics occurred among host and vector traits, which was a necessary simplification given that host and parasite phylogenies were independently simulated (i.e., cospeciation did not occur in host and parasite lineages). Because the simulated traits are realizations of a random process of evolution on the phylogeny, the actual degree of phylogenetic signal may differ from the user-specified value of λ. We tested whether the estimated λ for the simulated traits approximated user-defined λ and re-ran the simulation if this departed by more than 0.04 in simulations that varied a single parameter, and 0.06 in simulations that varied more than one parameter. To estimate λ in making this assessment, we used the phylosig function in phytools [71].

To implement selective extinction, we pruned host species on these networks based on the threat status trait, *h*_*2*._ Specifically, a proportion *p*_*x*_ of host species with highest values of *h*_*2*_ was pruned from the host tree, reducing the number of hosts (but keeping the number of vector species constant). When the λ transformation used to simulate *h*_*2*_ was set to equal 0, host extinction was random with respect to phylogenetic relatedness. As λ approached 1, the probability that sister species underwent co-extinction increased, with potentially greater effects on host community phylogenetic structure and on host-vector network structure. We varied *p*_*x*_ to assess whether increasing the proportion of extinctions in a network influences network structure. R code for the model functions is provided as Electronic S1 File.

### Host preferences and network structure

We used the simulated trait values *h*_*1*_ and *v* to determine host preferences by the vectors. The phylogenetic trait simulations resulted in different minima and maxima for each trait on the host and vector trees due to the random process of trait evolution. To make the host and vector traits comparable, we scaled both sets of simulated traits to range between 0 and 1. We then created a matrix of trait value differences, with columns representing host species and rows representing vectors, and took the absolute value of each difference. These differences were used as the basis for assigning preferences of a vector for a host, where higher preference was indicated by smaller difference between a host and its vector.

**Fig 2:**
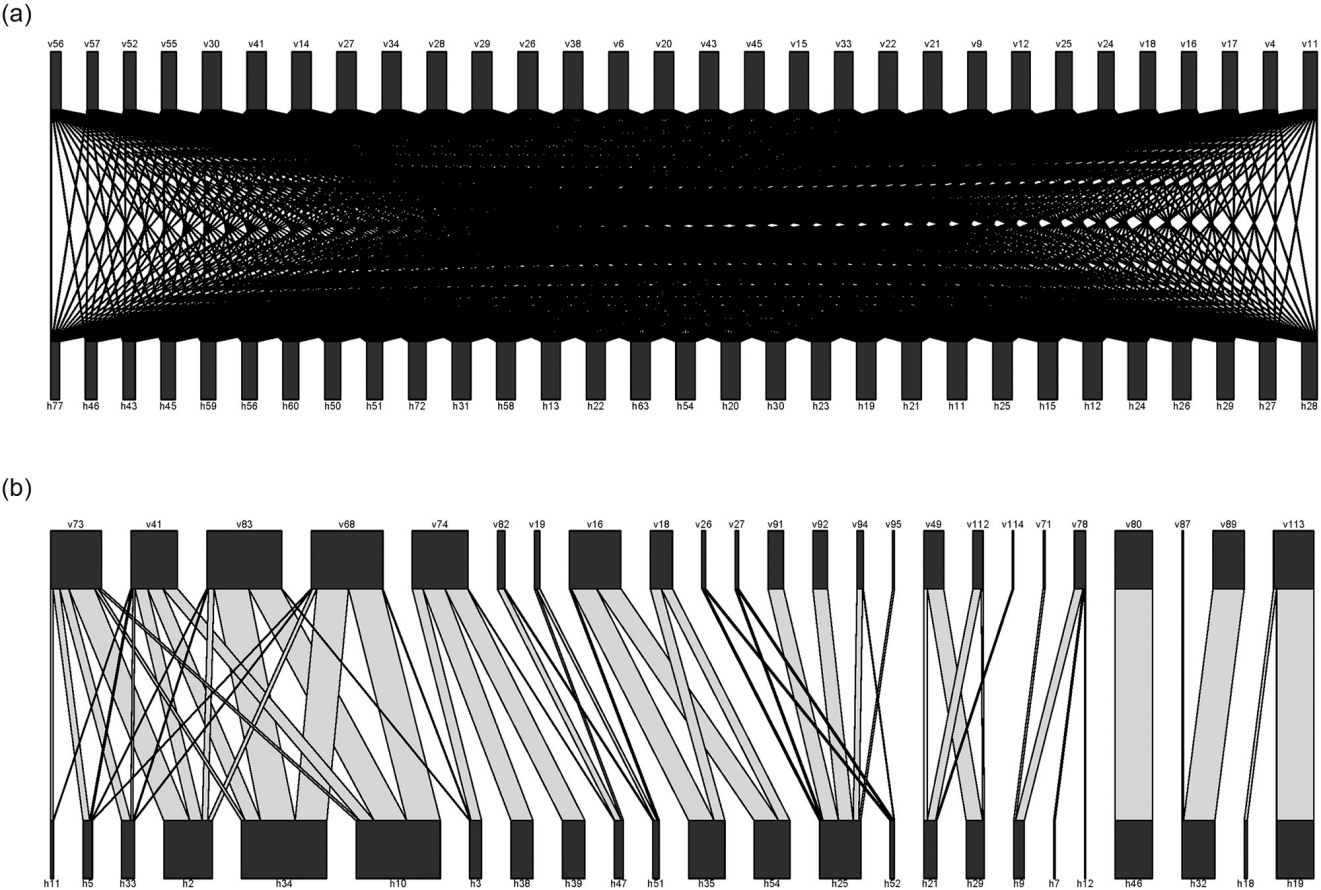
Bipartite networks for representative simulations with small and large values of host specificity (g). Width of bar reflects overall summed preference by vectors (top, taxa labeled starting with v) for hosts (bottom, taxa labelled starting with h), and plots show all preference scores between vectors and hosts that are 0.01 or greater (maximum value is always 1). The smallest version of g used was 0.021 (top plot), revealing generalist vectors. The largest value of g was 4.98, with more vectors specializing on only a few hosts.

We further assumed that the degree of host preference follows an exponential function, ${\left(1-X\right)}^{e^{g}}$, where *X* represents host preference and *g* is a parameter of host specificity, with higher values representing greater host specificity. To help visualize the effects of *g* on network structure, Fig 2 shows networks resulting from low *g* (left) and high *g* (right), revealing how increasing *g* results in isolation of communities (and thus, increased modularity). We allowed *g* to range from 0 to 5, as values greater than 5 tended to generate nonsensical ecological networks. Each score was divided by the sum of scores per vector to obtain proportional preferences (*P*) for a vector, resulting in a preference matrix.

From these simulated preference matrices that incorporated varying amounts of phylogenetic structure, we created bipartite networks based on simulated vector preferences for different hosts. We then used the R package bipartite [72] to estimate the number of modules on these bipartite networks and to calculate Newman’s Q as a measure of modularity. This measure is calculated based on weighted links between species, but not on abundance of those species. Q was calculated in the bipartite package [72] in R, based on the QuaBiMo algorithm [73]. We recognize that our measure of Q may be impacted by network size and other factors, such as the number of edges relative to total potential number of edges [61, 73]. We control for network size in our analyses, either by holding size constant across simulations or including it as a predictor in the statistical models in multivariate analyses. Our networks are often nearly fully connected (with strong heterogeneity in edge weight) due to how we calculated the preference matrix. We investigated possible null models to control for these effects but failed to find a convincing way to incorporate these potential biases into the calculation of modularity. Instead, in our multivariate models that analyzed *Q* before and after extinction, we also ran analyses that included three additional variables as predictors: “connectance” (i.e., realized proportion of possible links), “links per species” (i.e., mean number of links per species), and “linkage density” (i.e., weighted diversity of interactions per species), with statistics and definitions obtained from the bipartite package [72].

### Simulations of different bipartite networks

We first investigated the effects of individual parameters (bivariate analyses), holding all other parameters constant, with 200 simulations for each parameter that was varied. Ranges of parameter settings (and associated fixed values for other parameters) are given in Table 1. For the variable parameter in these simulation runs that held other variables constant, we selected parameters randomly from within 200 evenly spaced bins using the package lhs [74] in R [75].

**Table 1:** Community phylogenetic model and simulation parameters.

Parameters	Latin-Hypercube Sample (LHS) range	Fixed Values
Number of host species (*N*_*h*_)	25–60	40
Number of vector species (*N*_*v*_)	25–60	40
Birth rate in host lineages (*b*_*h*_)	Fixed	0.9
Death rate in host lineages (*d*_*h*_)	0.2–0.88	0.7
Birth rate in vector lineages (*b*_*v*_)	Fixed	0.9
Death rate in vector lineages (*d*_*v*_)	Fixed	0.7
Expected variance of simulated traits	Fixed	1
Host specialization index (*g*)	0–5	3
Pagel’s lambda (λ), for *h*_*1*_, *h*_*2*_ and *v* (i.e., λ_*h1*_, λ_*h2*_, λ_*v*_)	0–1	1
Proportion of species extinct (*p*_*x*_)	0.05–0.7	0.333

Next, to investigate a wider range of parameters simultaneously, we employed a multivariate analysis in which multiple model parameters were varied within a range of values (Table 1). Our parameters do not represent a specific system, but we used a reasonable range of parameters for a community of mosquito vectors and vertebrate hosts. For example, we chose to vary the number of hosts and vectors from 25 to 60, reasoning that most communities will have more than 25 hosts and most vector species would have well fewer than 60 hosts. In some cases, we adjusted parameter ranges if values produced nonsensical results (e.g., high values of *g* produced unstable networks, while *d*_*h*_ > 0.88 approached the fixed value of *b* = 0.9, causing long run times to achieve parameterized numbers of hosts and vectors, and resulting in atypical phylogenies). Parameter values (N = 1000) were drawn from a flat distribution to represent the input distribution using Latin hypercube sampling (LHS), which is more efficient than the random or full sampling [76–78]. The LHS was obtained with the R package lhs [74]. With the resulting sample of simulated host-parasite networks, we investigated network modularity and disease dynamics, including after extinction of hosts.

### Simulating infectious disease transmission

With the goal of ensuring that our outcomes involving network structure have epidemiological consequences, we generated a simple multi-host, multi-vector disease transmission model that was informed by Ross [79] and by other models that include multiple hosts [80, 81]. We intentionally implemented a simple model, as the goal was to assess how well network structural metrics predicted disease prevalence rather than to predict prevalence in a particular community. Each host and vector could be susceptible or infected, without any chance of recovery (an SI model). Disease transmission occurred stochastically over discrete time steps, as described by this set of equations:

1.
$H_{I,j}\left(t+1\right)={min\;\{H_{T},\;H}_{I,j}(t)+\frac{1}{H_{T}}\sum_{k=1}^{m}V_{I,k}\left(t\right)P_{j,k}\beta H_{S,\;j}(t)\}$

2.
$H_{S,j}\left(t\right)=H_{T}-H_{I,j}(t)$

3.
$V_{I,\;k}\left(t+1\right)={min\;\{V_{T},\;V}_{I,k}\left(t\right)+\frac{1}{H_{T}}\sum_{j=1}^{n}V_{S,k}(t)P_{j,k}\beta H_{I,j}(t)\}q$

4.
$V_{S,k}\left(t\right)=V_{T}-V_{I,k}(t)$

Where *H*_*I*,*j*_ and *H*_*S*,*j*_ represent the number of infected and susceptible individuals for a given host species *j*, *V*_*I*,*k*_ and *V*_*S*,*k*_ the number of infected and susceptible individuals for a given vector species *k*, *H*_*T*_ and *V*_*T*_ the total number of individuals in a host or vector species (both constants), *m* is the number of vector species, *n* is the number of host species, *P*_*j*,*k*_ is the proportional preference of vector species *k* for host species *j*, and *β* gives the probability that a contact between an infected individual and a susceptible individual results in an infection.

When running this model, host and vector populations were held constant and identical in size across species, with each host species assigned a population size of 100 and each vector species a population size of 200. While this diverges from the heterogeneity and larger population sizes observed in many real-world host-vector communities, these simplifying assumptions enabled us to focus on how network structure influences the transmission of vector-borne disease without the confounding effect of changes in relative abundance. We initiated disease transmission simulations by placing 1% of one host species in the infected category, with all other host species having no infected individuals. We set *β* to 0.05. To account for heterogeneity in connectivity among the different hosts, multiple simulations were run for each network such that the initial infections started in each different host species. We assumed that all vectors and host species respond similarly to infection, as complications with setting different transmission probabilities (*β*) would make the model overly complex and take the focus away from identifying the impact of vector-host modularity on disease transmission.

We assumed no added mortality due to disease, for both host and vector. Thus, when all communities in the network were connected, the disease eventually spreads to all individuals. We also ignored density effects, focusing on proportions of infected vectors rather than their density. As our primary response variable, we measured the prevalence in the community as a whole at time step 300. Although many different metrics could be obtained, the prevalence at a particular time point is intuitive and captures how effectively and quickly the simulated pathogen can spread, with higher modularity expected to reduce transmission efficiency in the network (resulting in lower prevalence). Computer code for implementing this model is included in the Electronic S1 File.

### Statistical analysis

Because we were interested in understanding how changes to vector-host communities impacts the structure of those communities, we examined the impact of various parameters (Table 1) on network modularity before (*Q*_*0*_) and after (*Q*_*1*_) host extinctions. We calculated this difference as Δ*Q* = *Q*_*0*_ –*Q*_*1*_, such that negative Δ*Q* indicates an increase in modularity after extinction. We also investigated the effects of these parameters on disease transmission through the changes in network structure. We used a simulation approach and analyzed our results with an information theoretic framework based on model averaging [82]. Specifically, we obtained an AIC for each of the possible models, and then averaged the estimated coefficients based on the AIC weight. In the initial bivariate tests, we focused on effects of a single parameter on modularity; in such cases, we compared the model with the variable to the null model, which included only an intercept. In other cases, we averaged across multiple models that included all combinations of parameters (e.g., when analyzing the LHS). We implemented full-model averaging, in which parameters that were not included in a model were set to 0 and included when averaging the coefficient estimates. These analyses were conducted in *R* [83] with the package MuMIn [84]. We provide standardized coefficients, standard errors (SE) of the coefficients, and a measure of importance, defined as the summed AIC weight of all models that included the variable (and thus having a maximum value of 1).

## Results

**Fig 3:**
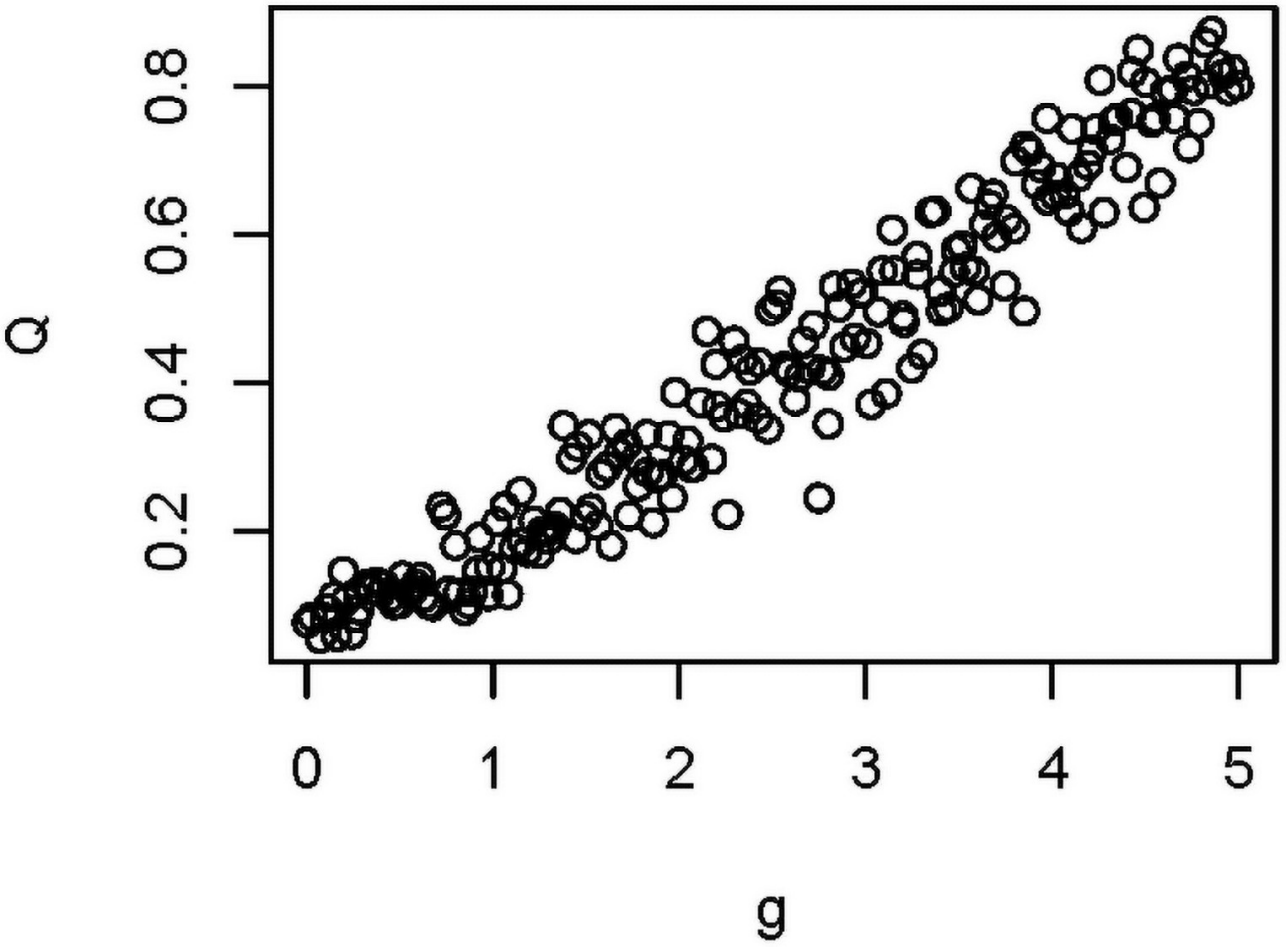
Effect of host specificity on Q. Simulations in which g was varied, holding all other parameters constant at their values in Table 1. A strong positive association was found (see text and Table 4).

We first examined the effects of variables individually on modularity calculated before extinction (*Q*_*0*_) and again after extinction (*Q*_*1*_), holding other variables constant. Of the eight variables in Table 1, the extent of vector specialization (*g)* showed the strongest effect on modularity (Fig 3), with *Q* increasing with increasing *g*. This effect is driven by substantially greater network structure at higher *g* (Fig 2). We found strong support for including *g* in a model predicting *Q*_*0*_ (b = 0.97; AICc = -574.7, vs. AICc = -6.25 in a model with only the intercept, i.e. ΔAICc = 568, importance = 1.0), with nearly identical results for *Q*_*1*_.

**Fig 4:**
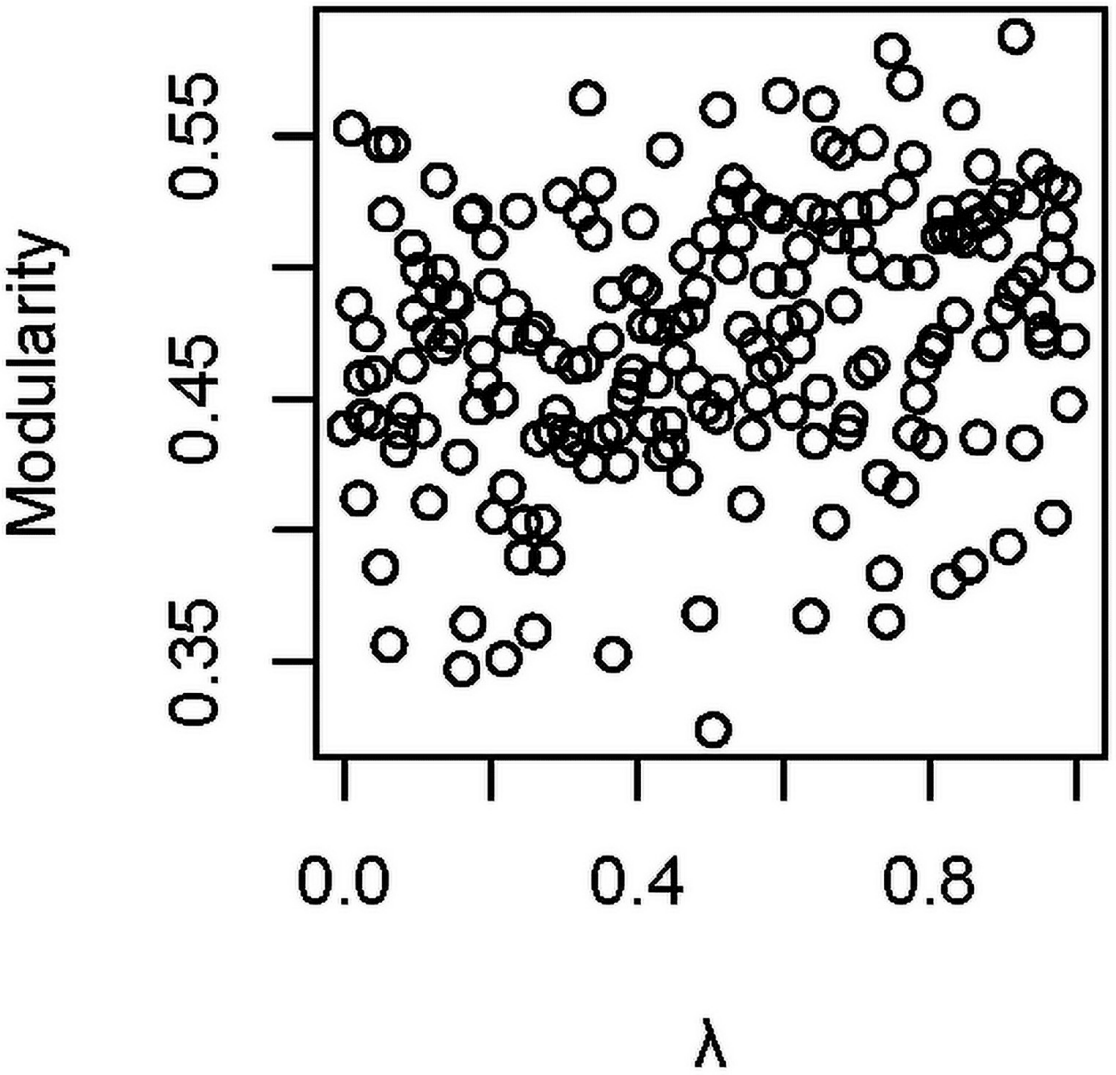
Association between phylogenetic signal (λ) and modularity (*Q*_*0*_). Results are from bivariate tests, with λ_*h1*_ = λ_*v*_.

The proportion of hosts undergoing extinction (*p*_*x*_) also showed a positive association with *Q*_*1*_ (b = 0.32, SE = 0.068, ΔAICc = 18.9, importance = 1.0), while the total number of vectors (*v*) in the simulation negatively influenced *Q*_*0*_ (b = -0.26, SE = 0.070, ΔAIC = 12.1, importance = 1), with somewhat weaker effects on *Q*_*1*_ (b = -0.15, SE = 0.087, ΔAIC = 3.69, importance = 0.86). The death rate in host lineages (*d*_*h*_) showed no evidence of an association with *Q*_*0*_ (model averaged effect on *Q*_*0*_: b = 0.014, SE = 0.044, ΔAICc = 1.65, importance = 0.3; similar results were obtained in analyses of *Q*_*1*_). Variation in phylogenetic signal (λ) for host and vector traits, including extinction risk (i.e., *h*_*1*_, *h*_*2*_ and *v*), also appeared to be largely unrelated to *Q*_*0*_ (importance values were all less than 0.50) in these bivariate tests. However, when we ran simulations in which λ for host and vector traits was equal (i.e., λ_*h1*_ = λ_*v*_, with λ varying from 0 to 1), we found evidence for positive associations between λ and modularity (b = 0.234, SE = 0.072, ΔAICc = 9.41, importance = 0.99; Fig 4).

**Fig 5:**
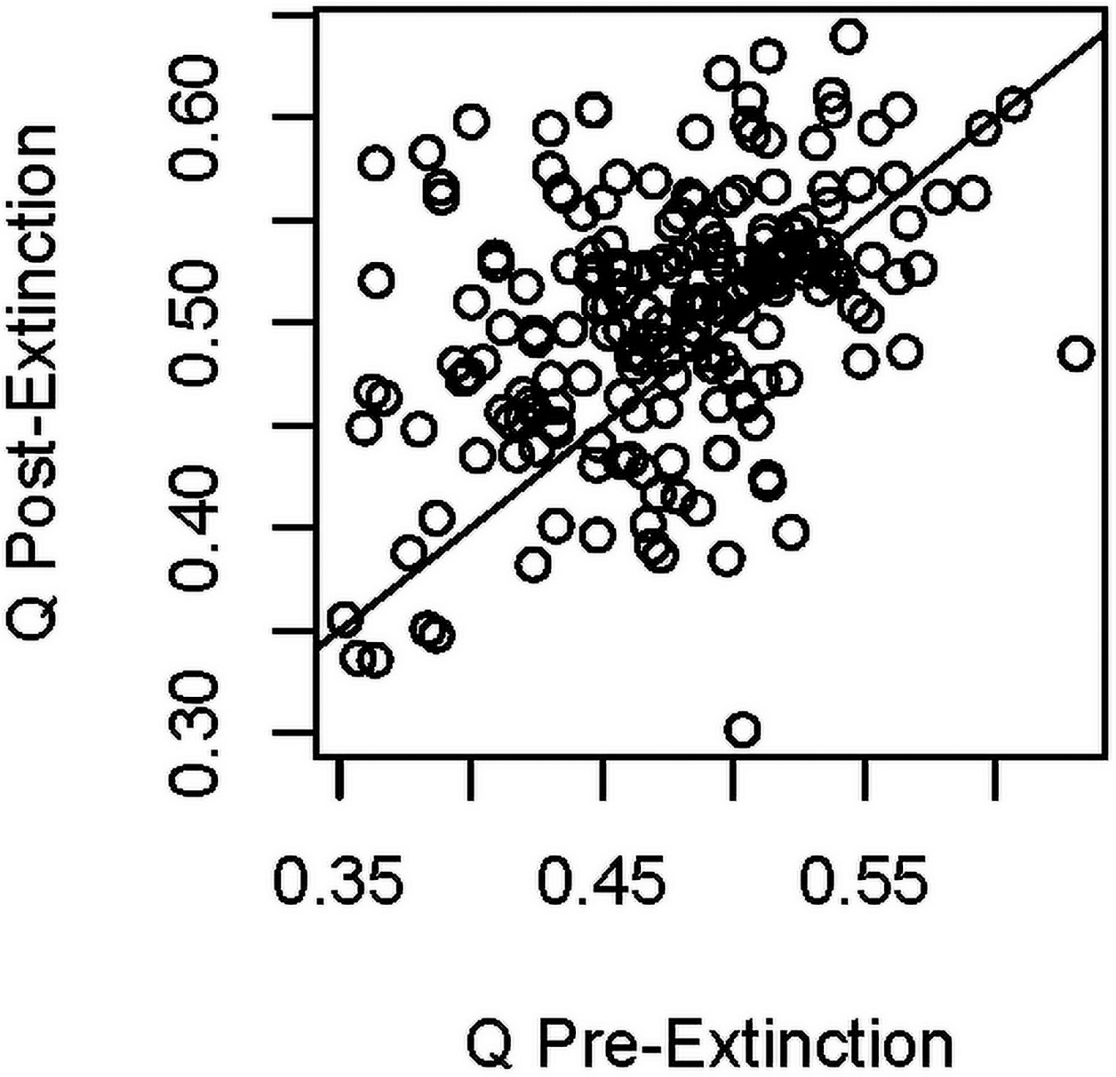
Modularity before and after extinction. Line shows a slope of 1 through the intercept; thus, positive residuals indicate higher *Q* after extinction (i.e., *Q*_*1*_ > *Q*_*0*_, and Δ*Q* is negative). Results here are from simulations that varied phylogenetic signal in the vector trait (*v*), but similar results were found in other simulations.

For each of these bivariate models, we also investigated how extinction affected modularity by calculating the difference in modularity before and after extinction (Δ*Q* = *Q*_*0*_ –*Q*_*1*_, with *p*_*x*_ = 0.333 for all simulations, except when varying *p*_*x*_). We found that modularity generally increased following extinction (Table 2, Fig 5). Moreover, Δ*Q* was influenced by *p*_*x*_, with a higher proportion of the clade going extinct leading to a higher value of modularity post-extinction (Table 2). Although weaker, we found some evidence that increases in host specificity (*g*) also negatively influenced Δ*Q*.

**Table 2:** Effects of individual variables on Δ*Q* in bivariate analyses.

Analysis	Mean Δ*Q*	Effect of Variable (b ± SE)	ΔAICc	Importance
*N*_*h*_	-0.026	0.0036 (0.037)	2.02	0.27
*N*_*v*_	-0.018	-0.017 (0.048)	1.48	0.32
*d*_*h*_	-0.024	0.0016 (0.037)	2.05	0.26
*g*	-0.016	-0.15 (0.086)	4.03	0.88
*λ*_*h1*_	-0.030	0.047 (0.069)	0.17	0.48
*λ*_*h2*_	-0.032	0.021 (0.051)	1.32	0.34
*λ*_*v*_	-0.027	0.0034 (0.037)	2.03	0.27
*p*_*x*_	-0.032	-0.29 (0.068)	14.9	1.00

Notes: Definitions of variables are provided in Table 1. Coefficients are standardized, and ΔAICc refers to the change in AIC in the bivariate model with that variable, as compared to a null with an intercept only. These are outcomes of eight individual analyses that vary the parameter shown, with all other variables fixed as in Table 1. **Δ***Q* is calculated as *Q*_*0*_ –*Q*_*1*_, such that negative values indicate positive changes in modularity following extinction. Importance refers to the summed AIC weights of all models that included the variable (and thus it has a maximum value of 1).

**Fig 6:**
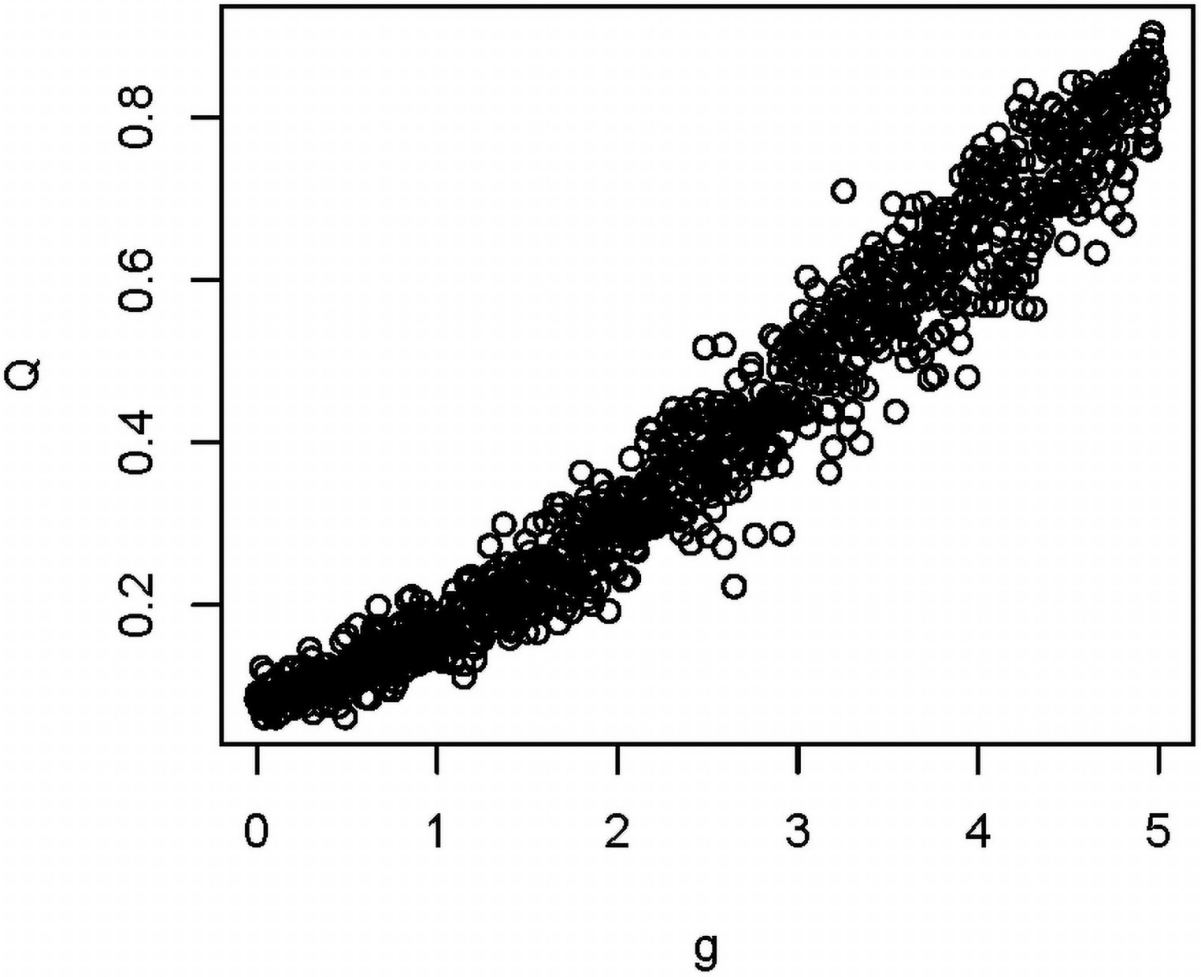
Relationship between host specificity (*g*) and modularity (*Q*). Data come from the 1000 simulations using the Latin hypercube sample (LHS). The measure of modularity is pre-extinction (i.e., *Q*_*0*_).

**Fig 7:**
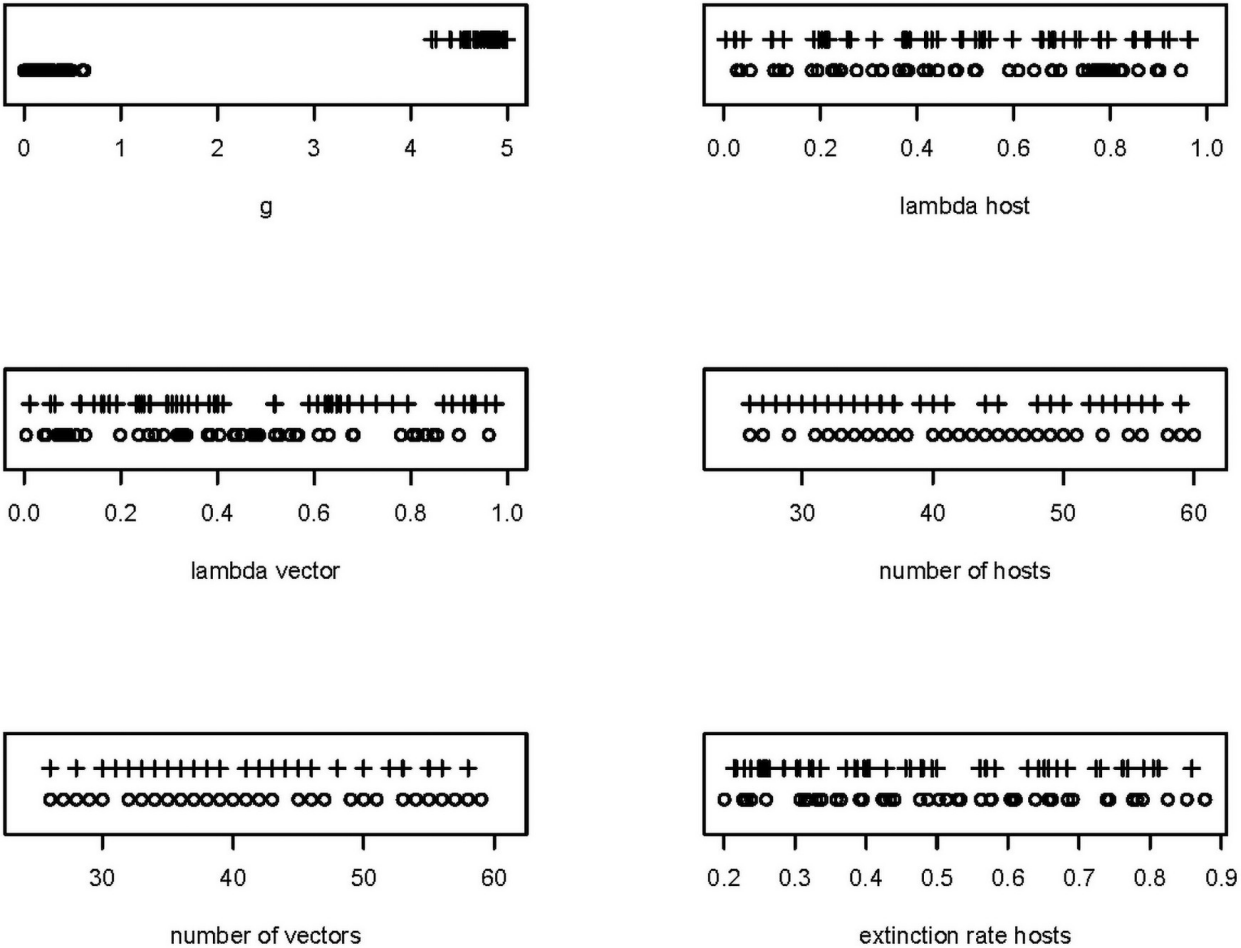
Effects of variables on maximum and minimum values of *Q*. For each variable, we indicate the 50 highest (+ symbol) and 50 lowest (circles) values of *Q*_*0*_ from simulations conducted with the LHS. This provides a way to assess whether high extreme output values are consistently associated with particularly high or low values of each variable.

Next, we generated a LHS of 1000 sets of simulation parameters, varying eight of the parameters as summarized in Table 1. We found that of these variables, *g*, λ_*h1*_, λ_*v*_, and the network characteristic “links per species” had highest importance in predicting *Q*_*0*_ (Table 3), while *g* and the network characteristic “linkage density” had highest importance for predicting *Q*_*1*_, with weaker effects for λ (Table 4). The shape of the effect of *g* on *Q*_*0*_ and *Q*_*1*_ was also similar to that found when investigating individual variables, although with a slightly more logistic shape (Fig 6, for *Q*_*0*_). Looking at the highest and lowest *Q*_*0*_ and Δ*Q* outputs, *g* appeared to consistently impact both variables: the highest values of *Q*_*0*_ were always associated with high *g* (and conversely, lowest values were always associated with low *g*, Fig 7). In addition, statistical analysis revealed positive effects of *λ*_*h1*_ and weaker effects for *λ*_*v*_ (Table 3).

**Table 3:** Model averaged effects of six variables on modularity (pre-extinction).

Analysis	Effect of Variable (b ± SE)	Importance
*g*	0.96 (0.025)	1.00
*λ*_*h1*_	0.019 (0.0071)	0.98
*λ*_*v*_	0.014 (0.0078)	0.89
*links per species*	-0.054 (0.032)	0.83
*linkage density*	-0.016 (0.025)	0.46
*N*_*v*_	-0.012 (0.022)	0.42
*d*_*h*_	-0.017 (0.080)	0.35
*N*_*h*_	0.0040 (0.080)	0.34
*connectance*	-0.0044 (0.013)	0.32

Statistical model: *Q*_*0*_ **~** *d*_*h*_ **+** *g* **+** *λ*_*h1*_ + *λ*_*v*_ + *N*_*h*_ + *N*_*v*_ + *connectance* + *links per species* + *linkage density*, leaving out *p*_*x*_ and *λ*_*h2*_ because they are not relevant to pre-extinction modularity. Results are ordered from highest “importance” to lowest “importance,” where importance refers to the summed AIC weights of all models that included the variable (and thus it has a maximum value of 1).

**Table 4:** Model averaged effects of eight variables on modularity (post-extinction).

Analysis	Effect of Variable (b ± SE)	Importance
*g*	0.84 (0.024)	1.00
*linkage density*	-0.15 (0.025)	1.00
*connectance*	-0.016 (0.017)	0.65
*λ*_*h1*_	0.0064 (0.0075)	0.58
*λ*_*h2*_	0.0063 (0.0074)	0.57
*links per species*	-0.011 (0.017)	0.48
*λ*_*v*_	0.0044 (0.0066)	0.48
*N*_*v*_	-0.0042 (0.0089)	0.40
*d*_*h*_	-0.0098 (0.079)	0.39
*N*_*h*_	0.0017 (0.079)	0.38
*p*_*x*_	-0.00054 (0.011)	0.38

Statistical model: *Q*_***1***_ **~** *d*_*h*_ **+** *g* **+** *λ*_*h1*_ + *λ*_*v*_ + *N*_*h*_ + *N*_*v*_ + *p*_*x*_ + *λ*_*h2*_ + *connectance* + *links per species* + *linkage density*. Results are ordered from highest “importance” to lowest “importance,” where importance refers to the summed AIC weights of all models that included the variable (and thus it has a maximum value of 1).

**Fig 8:**
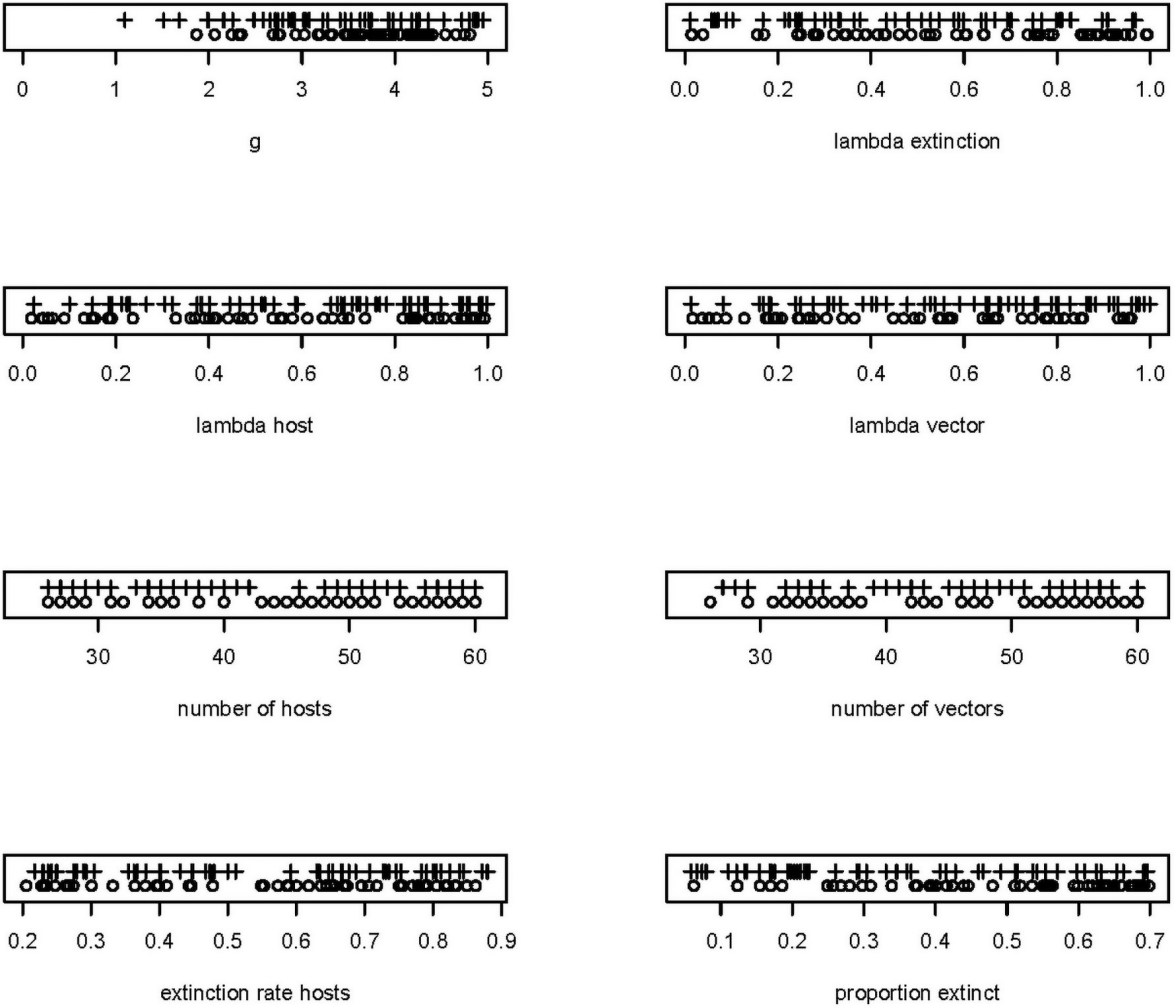
Extreme values of pre-extinction modularity—Post-extinction modularity (ΔQ) in relation to eight parameters in the LHS. For each variable, we indicate the 50 highest (+ symbol) and 50 lowest (circles) values of ΔQ from simulations conducted with the LHS. This provides a way to assess whether high extreme output values are consistently associated with particularly high or low values of each variable. ΔQ is calculated as *Q0* –*Q1*, such that negative values indicate positive changes in modularity following extinction.

When investigating predictors of Δ*Q* in the LHS, we again found a modestly higher *Q* in the post-extinction sample, i.e. Δ*Q* was, on average, negative (= -0.018). In model averaging with Δ*Q* as the response variable, the effect of *p*_*x*_ was again striking, with a higher proportion of hosts going extinct leading to higher modularity (b = -0.24, SE = 0.03, importance = 1). Similarly, increases in *g* led to higher modularity in the post-extinction networks (b = -0.16, SE = 0.030, importance = 1). All other variables had importance scores of 0.6 or lower. Overall, results involving Δ*Q* were visually weaker in analyses of the post-extinction sample (Fig 8).

**Fig 9:**
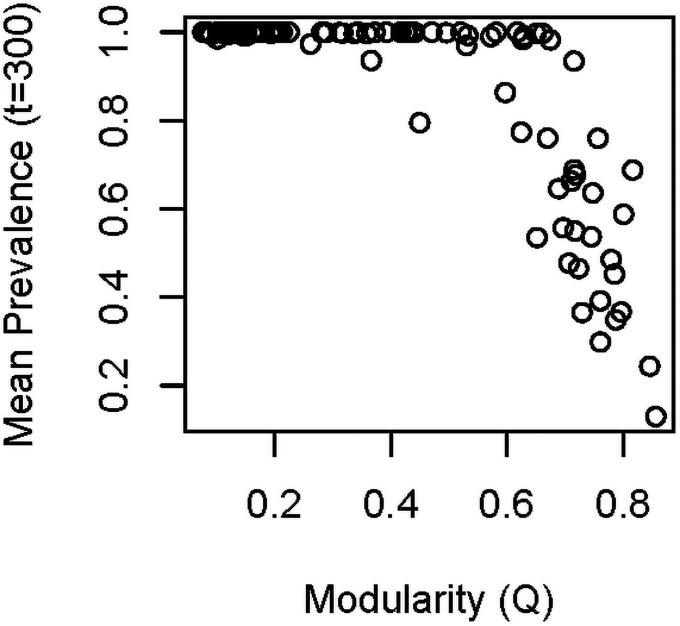
Prevalence of infection in the simulated communities in relation to host modularity. We ran simulations on 100 networks from a Latin Hypercube sample, using the S-I model described in the Methods. Results here represent prevalence at time step 300, averaged across simulations in which the disease was initiated in different hosts.

Finally, we simulated disease transmission on the networks to investigate whether our measures of modularity are likely to have epidemiological implications. To reiterate, our purpose was to confirm that modularity influences disease prevalence in the simulated communities, rather than to generate specific predicted disease outcomes in these communities. We generated 100 LHS networks as above, and ran simulations starting the infection on each of the different hosts. At time step 300, we found that prevalence of 100% was common when *Q* was less than about 0.4, but rarely at 100% when *Q* was above 0.6 (Fig 9). We fit a polynomial model to the data that included number of hosts and number of vectors as additional predictors (i.e., prevalence_300_ ~ *Q* + *Q*^*2*^ + *N*_*h*_ + *N*_*v*_). The polynomial model was supported over a linear model and a null model that included only number of hosts and number of vectors (AIC_poly_ = -191.2, AIC_linear_ = -108.9, AIC_null_ = -19.1). In model averaging, all terms in the polynomial model received importance scores of 1.

## Discussion

Of the variables that we investigated, the degree of host specificity by vectors had the strongest effects on modularity. When host specificity was high (resulting from vectors having strong preferences for fewer hosts), modularity in the resulting ecological networks increased substantially. This makes sense, as high host specificity would lead the network to be more structured (Fig 2), with vectors preferring only a handful of hosts and showing less overlap among other vectors.

We anticipated a consistent effect of vector specialization (*g)* on modularity in advance and used this expectation as a point of comparison to other, less easily predicted effects. Changes in vector specialization had strong and consistent effects on modularity, whereas variation in other parameters had less consistent effects. With regard to changes in the host community, for example, host extinction tended to increase network modularity, although this was not found in all simulations (Fig 5). The effects of extinction on modularity in the simulations increased with increasing proportion of host species that went extinct. These effects likely occurred because extinction tends to prune closely related species from the tree, resulting in stronger phylogenetic clustering of host preferences and parasite traits (and thus greater modularity). Coupled with our simulations showing that higher modularity decreases disease prevalence (Fig 7), these findings suggest that, all else equal, communities that have experienced high rates of extinction (and thus contain only a subset of the original species) may have lower disease prevalence than intact communities, and that increasing the proportions of species going extinct will cause greater reductions in prevalence. This may result in increased coextinction of hosts and parasites [85].

Of course, the effects of disturbance on communities are unlikely to be as simplistic as simulated in our study, with no within-species changes in abundance following extinctions and no species additions, and also with no variation in transmission probability based on host identity and no variation in competence among species. Nonetheless, our findings suggest that one effect of host species loss is to *reduce* disease risk through changes to host-vector transmission networks. In real communities, these effects may often be offset by changes in disease risk due to other factors, including in relation to heterogeneity in competence that may covary with extinction risk. These factors could result in a dilution effect [41, 86] or provide opportunities for invasion by particularly competent hosts or highly efficient vectors [13, 87]. The altered networks may also select for different transmission characteristics of pathogens, including use of different vectors.

More surprisingly, we only found effects of phylogenetic signal in host and vector traits in simulations where phylogenetic signal of host and vector traits were similar, or when these traits were both allowed to vary (i.e., in analyses of *Q*_*0*_ in the LHS). We anticipated that when more closely related species of hosts and vectors share phenotypic traits and preferences, respectively, this would lead to clustering of interactions similar to the effects of vector specificity (*g*), resulting in higher modularity. This could result from convergent physiological (e.g. adaptation to digesting certain blood types), behavioral (attraction to host specific signals) or habitat adaptations within a clade. However, it appears that these effects are weaker than expected, and perhaps depend on the specifics of other trait linkages to phylogeny.

The model used here is theoretical, but could be parameterized for specific host-vector-parasite systems, or studied using data on host-vector associations to generate empirical bipartite networks. Some data exist on host-vector associations, including even data on the infectious diseases transmitted in those ecological associations [88]. Similarly, data is increasingly available on mosquito-host associations, including via analyses of blood meals [89–91]. Clearly, not all parameters in our model are easily quantified in empirical systems, but many of them could be investigated empirically in future research. Connecting our theoretical study to empirical data can improve predictions for likely vector-host-pathogen associations that threaten human health, as has been suggested by work predicting Zika virus vectors in North America [92].

Successful parameterization of this model to fit real communities would have multiple benefits. Loss of host species from a community following anthropogenic disturbance has the potential to radically transform networks of host-vector connections, and thus to alter disease transmission. Given the extremely high rate at which native host communities are now changing–in diversity, composition and phylogenetic structure [17, 93–97], especially in areas with high human disturbance [98]–this creates strong potential for changes in zoonotic disease dynamics and emergence of disease [32, 36, 41, 99]. Based on the tremendous burden that vector borne diseases exert on humans, livestock and wildlife, particularly in tropical countries, this could have strong impacts on global health [38]. Some work, both experimental and in natural systems, has suggested that changes in phylogenetic community structure, typical in anthropogenically disturbed sites, may be particularly important in driving disease transmission dynamics [32, 36, 99, 100]. Our investigation of these dynamics by simulating host-vector bipartite networks in an explicitly phylogenetic framework elucidates the effects of both phylogeny and host specificity on these networks, and the impacts of evolved traits that covary with extinction risk (e.g. life history traits) on changes in host-vector networks following loss of species from the system.

If the goal was to more explicitly model disease transmission (rather than simply network structure) in a real-world host-vector system, the model could also include variation in host and vector abundance (which here was assumed to be constant). With such a model, one could perturb the system through specific, real-world changes in host or vector abundance to assess how extinction of hosts impacts network structure and pathogen transmission. Other future directions would also be useful. Here, for example, we focused on structural properties of networks; effects on node importance (centrality) could be investigated in the future. Similarly, the model could incorporate coevolutionary dynamics that might involve, for example, associations between network structure and extinction or cospeciation of host and vector lineages, or richer investigations of how different extinction processes directly affect disease prevalence in the simulated networks. In addition, the model could be used to investigate effects of introduced vectors or hosts on disease spread in ecological networks. Similarly, extinction of vectors could be investigated in this framework.

In conclusion, our model suggests that host extinction from ecological communities will often–but not always–lead to increased network modularity, likely reducing disease transmission, although these effects will be balanced by other effects of disturbed communities that may increase disease transmission. The degree of vector specificity on particular hosts has a strong impact on modularity, with some indications that the level of phylogenetic signal in host and vector traits can impact the modularity of host-parasite ecological networks. Important steps for the future are to use similar models to assess the relative effects of additional mechanisms that may amplify disease transmission in real-world disturbed systems, such as the loss of less competent hosts during extinction (i.e., the dilution effect), and to investigate how changes in host community composition affect the abundance of the remaining community members. In addition, it will be important to also consider vector communities in the context of human disturbance [12], and how changes to those communities influence disease transmission.

## Supporting information

S1 File (R)
